# Complete Genome Sequences of Cellvibrio japonicus Strains with Improved Growth When Using α-Diglucosides

**DOI:** 10.1128/MRA.01077-19

**Published:** 2019-10-31

**Authors:** Cecelia A. Garcia, Jackson A. Narrett, Jeffrey G. Gardner

**Affiliations:** a Department of Biological Sciences, University of Maryland–Baltimore County, Baltimore, Maryland, USA; University of Southern California

## Abstract

Cellvibrio japonicus is a saprophytic bacterium that has been studied for its substantial carbohydrate degradation capability. We announce the genome sequences of three strains with improved growth characteristics when utilizing α-diglucosides. These data provide additional insight into the metabolic flexibility of a biotechnologically relevant bacterium.

## ANNOUNCEMENT

**FIG 1:**
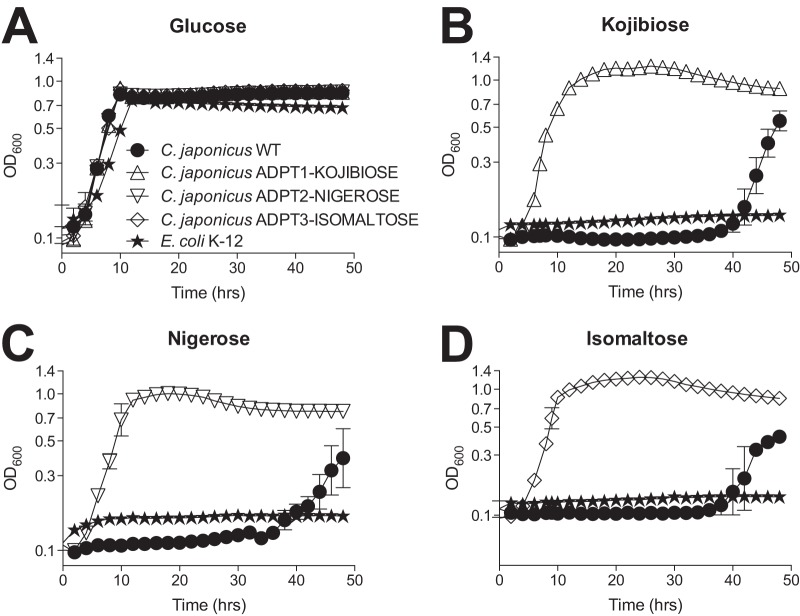
Growth phenotypes of wild-type *C. japonicus* and strains selected for improved utilization of α-diglucosides as the sole carbon source. (A) All *C. japonicus* strains were able to grow using glucose at comparable levels. (B to D) When kojibiose (B), nigerose (C), or isomaltose (D) was used as the sole carbon source, wild-type *C. japonicus* (closed circles) was unable to grow for over 30 h. Conversely, the isolated strains (open symbols) began to grow immediately in their respective α-diglucoside, had a high growth rate, and obtained a higher maximum level of growth compared to that of the wild type. For all α-diglucoside growth experiments, Escherichia coli K-12 (closed stars) was used as a negative-control strain.

Cellvibrio japonicus Ueda107 (NCIMB 10462) is a Gram-negative bacterium that was isolated from Japanese soil in 1952 (1–3). *C. japonicus* possesses over 200 genes that encode carbohydrate-active enzymes (CAZymes), many of which have been studied for applications in renewable energy (4–6). While the bacterium exhibits a robust carbohydrate utilization metabolism, the Ueda107 strain exhibits an extended lag phase when using α-diglucosides (Fig. 1). To select for *C. japonicus* strains with improved growth when using kojibiose, nigerose, or isomaltose as a sole carbon source, we grew the wild-type bacterium for 48 h in a single disaccharide (0.5% [wt/vol]) using microplate growth parameters identical to those previously published (7, 8). After 48 h, the cultures were reinoculated (1:100 dilution) back into the same carbon source, which resulted in growth phenotypes similar to those observed using glucose (Fig. 1). After 48 h of growth, 100 μl of each of the three reinoculated strains was individually recovered, mixed with 100 μl of 50% sterile glycerol, and frozen at –80°C for long-term storage.

To obtain sufficient cell mass for DNA extraction, we used the protocols published for *C. japonicus* Ueda107 RNA sequencing (RNA-seq) analysis (9–12). Genomic DNA was then extracted using a QIAamp high-throughput (HT) DNA kit (Qiagen, Hilden, Germany) and fragmented with an LE220 ultrasonicator (Covaris, Inc., Woburn, MA), which had an insert size between 253 and 267 bp. Genomic libraries were constructed using a Nextera XT library preparation kit (Illumina, Inc., San Diego, CA), following the manufacturer’s instructions. DNA quality and quantity were determined using either Qubit fluorometric quantification (Thermo, Waltham, MA) or TapeStation DNA electrophoresis (Agilent, Santa Clara, CA) when appropriate, with the merits of each previously described (13). A MiSeq platform with 2 × 150-bp paired-end read sequencing was used to generate reads for each strain (Illumina, Inc.). Raw sequence data (.bcl files) generated from the Illumina MiSeq platform were converted into FASTQ files and demultiplexed using the Illumina bcl2fastq 2.17 software. Default software parameters were used, with the exception that one mismatch was allowed for index sequence identification. Adapter sequences were trimmed during this process, which resulted in an average read length of 125 bp. The mean Q score for all three strains was approximately 36%, and the processed reads were mapped and assembled using CLC Genomics Workbench v10.0, with the default software settings (Qiagen), against the published *C. japonicus* Ueda107 reference genome (GenBank accession number NC_010995). In all cases, 100% of the *C. japonicus* reference genome (4,576,573 bp) was covered, and the average G+C content of all three strains was 52%. The strain isolated from kojibiose medium (*C. japonicus* ADPT1-KOJIBIOSE) had 3,379,938 mapped reads (107-fold coverage), with a complete genome size of 4,576,591 bp (3,687 total genes). The strain isolated from nigerose medium (*C. japonicus* ADPT2-NIGEROSE) had 3,281,977 mapped reads (104-fold coverage), with a complete genome size of 4,576,586 bp (3,688 total genes). The strain isolated from isomaltose medium (C. *japonicus* ADPT3-ISOMALTOSE) had 3,204,888 mapped reads (101-fold coverage), with a complete genome size of 4,576,591 bp (3,687 total genes). All three strains were annotated using the NCBI Prokaryotic Genome Annotation Pipeline (14). Given the similarity between the strains, a full comparative analysis may provide greater insight into the mechanisms for using α-diglucosides.

### Data availability.

The unique BioProject identifier for the entire study is PRJNA561085. The genome sequences were deposited in NCBI GenBank under accession numbers CP043304, CP043305, and CP043306. The raw data were deposited in the NCBI SRA under experiment numbers SRX6736265, SRX6736266, and SRX6736267.
